# Association Between Diabetes and Site-Specific Cancer Risk: A Population-Based Cohort Study on the Differential Role of Metabolic Profiles

**DOI:** 10.1155/jdr/1271189

**Published:** 2025-08-11

**Authors:** Sarah Tsz Yui Yau, Chi Tim Hung, Eman Yee Man Leung, Ka Chun Chong, Albert Lee, Eng Kiong Yeoh

**Affiliations:** JC School of Public Health and Primary Care, The Chinese University of Hong Kong, Hong Kong SAR, China

## Abstract

This study is aimed at investigating (i) whether diabetes is associated with each site-specific cancer and (ii) whether metabolic factors (lipids and liver enzyme) are differentially linked to different site-specific cancers by diabetes status. A retrospective cohort study was performed using electronic health records of Hong Kong. Patients who utilized public healthcare services between the year 1997 and 2021 with complete laboratory records and no cancer history were included. Patients were followed up until December 31, 2021. The associations with each site-specific cancer (colon and rectum, liver, pancreas, bladder, kidney, stomach, and lung) were assessed using Cox regression. A total of 197,906 patients were included. Patients with primarily Type 2 diabetes had a higher risk of developing liver and pancreatic cancers (aHRs for liver: 1.39, 95% CI = 1.11–1.75; pancreas: 2.04, 95% CI = 1.40–2.96) when compared to those without diabetes. Each 1 mmol/L increase in fasting glucose was associated with a 4% and 8% elevated risk of developing liver and pancreatic cancers, respectively. In general, lower lipids were linked to an increased risk of several malignancies (liver, pancreas, kidney, and stomach). In conclusion, diabetes is associated with an elevated risk of liver and pancreatic cancers. Baseline lipids and liver enzyme could be differentially linked to the risk of cancers at different organ sites by diabetes status.

## 1. Introduction

Cancer is one of the leading causes of premature death worldwide [1]. Concurrently, the global prevalence of diabetes is estimated at 10.5% [2]. With the rising trends of cancer mortality and diabetes prevalence [1, 2], cancer and diabetes conditions often coexist among the same patients more frequently than by chance [3].

Prior research has shown that patients with diabetes are at higher risk of developing cancer at several sites, including the liver, pancreas, colon, and rectum, as well as bladder [3]. The associations between diabetes and cancers of the liver and pancreas appear to be greatest in magnitude [3, 4]. On the other hand, the relationships between diabetes and cancers of the kidney and stomach remain less conclusive [3, 5, 6]. No association between diabetes and lung cancer has been demonstrated [3, 7]. Nevertheless, a recent meta-analysis found that evidence for the association between diabetes and colorectal cancer remains most robust (comparing to liver and pancreatic cancers), after accounting for potential bias [8].

Several lines of research have explored the associations between metabolic factors (glucose, lipids, and liver enzyme) and cancer risk. Two recent meta-analyses showed that fasting glucose is independently positively linked to the risk of pancreatic [9] and liver cancers [10]. A cohort study examining the relationships between glycated haemoglobin and the risk of 16 cancer sites found that glycated haemoglobin is only independently positively associated with the risk of pancreas, but not colorectal, bladder, kidney, gastric, or lung cancers [11]. Moreover, previous studies investigating the associations between lipids and cancer risk produced mixed findings [12, 13]. Given that lower levels of triglycerides and low-density lipoprotein (LDL) cholesterol and a higher level of high-density lipoprotein (HDL) cholesterol are linked to positive cardiovascular outcomes, it would be intuitively expected that the same lipid profile would be associated with favorable cancer incidence outcomes. Nevertheless, some epidemiological studies reported inverse associations between lipids and cancer risk [14, 15]. Furthermore, despite alanine aminotransferase (ALT) being considered an indicator of liver damage [16], several studies reported that ALT could also be linked to other digestive cancers [17]. In addition, studies examining the relationships between ALT and overall cancer risk produced opposite findings [17]. However, metabolic profile itself may potentially serve as markers of underlying health conditions. For example, while hyperlipidemia and elevated ALT may indicate potential cardiovascular conditions and liver diseases, respectively, lower levels of lipids and ALT may also be observed during illnesses [18] and aging [19].

While past research has shown the frequent co-occurrence of diabetes and cancer among the same patients [3], there is a lack of individual studies systematically examining the associations between diabetes and the risk of different site-specific cancers. Moreover, studies on the associations between metabolic factors (fasting glucose, lipids, and liver enzyme) and site-specific cancer risk remain lacking. In addition, past studies on the associations between lipids, ALT, and site-specific cancer risk reported mixed findings. Furthermore, it remains unclear whether altered metabolic profiles are associated with differential site-specific cancer risk and whether different patterns exist across diabetes and nondiabetes populations.

The primary objective of this study is to examine whether diabetes is associated with each site-specific cancer. The secondary objective is to explore whether metabolic factors (fasting glucose, lipids, and liver enzyme) are differentially linked to different site-specific cancers, and the patterns differ by diabetes status.

## 2. Methods

### 2.1. Study Design and Study Population

A retrospective cohort study was conducted using territory-wide electronic health records of Hong Kong. The Hospital Authority (HA) is a statutory body providing public healthcare services and maintains a centralized clinical data repository on patient demographics, disease diagnoses, prescription records, laboratory measurements, radiology reports, and clinical notes. Data used in this study were linked to death records from the Immigration Department. Disease diagnoses were coded according to the International Classification of Disease 10^th^ revision (ICD-10) or the International Classification of Primary Care 2^nd^ edition (ICPC-2). Prior research [20] has shown that data recording in the electronic health records system of Hong Kong is relatively comprehensive and accurate. Individual-level data across datasets were linked via pseudonymous identifiers. Data were accessed via HA Data Collaboration Lab.

### 2.2. Patients

**Figure 1:**
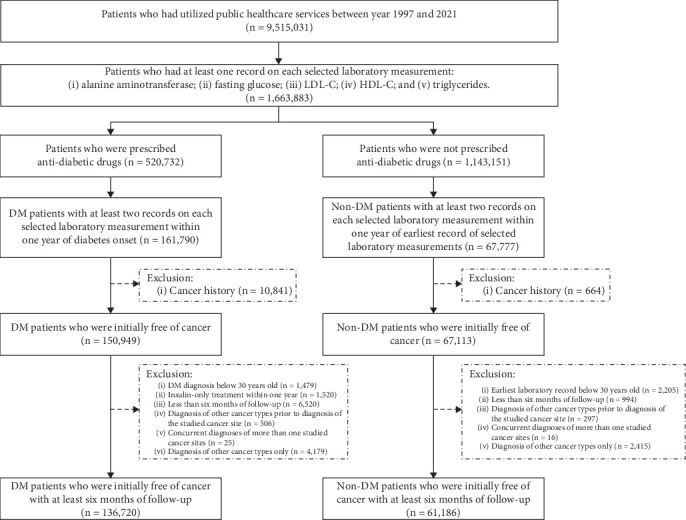
Flow chart of patient selection. Abbreviations: DM, diabetes mellitus; HDL-C, high-density lipoprotein cholesterol; LDL-C, low-density lipoprotein cholesterol.

Patients who had utilized public healthcare services between the years 1997 and 2021 were initially identified. Those who had at least one record on each selected laboratory measurement (ALT, fasting glucose, LDL cholesterol, HDL cholesterol, and triglycerides) were included. Records of the use of antidiabetic drugs were extracted to identify the diabetes status of patients. The diagnosis of diabetes was made by doctors when patients had two abnormal test results of plasma glucose and presentation of clinical symptoms. Patients with primarily Type 2 diabetes (*n* = 520,732) were followed up since the onset of diabetes or initiation of any antidiabetic drugs. Those with (i) less than two records on any of the laboratory measurements within 1 year of diabetes onset or (ii) a history of malignancy prior to diabetes onset were excluded. Patients without diabetes (*n* = 1,143,151) were followed up since the earliest record of selected laboratory measurements. Those with (i) less than two records on any of the laboratory measurements within 1 year of the earliest record time or (ii) a history of malignancy prior to laboratory measurements were excluded. To exclude possible cases of Type 1 diabetes, patients who received (i) a diagnosis of diabetes below 30 years old [21, 22] or (ii) a diagnosis of diabetes below 60 years old [21] and insulin treatment within 1 year of diabetes diagnosis (but no other antidiabetic drugs during study period) were further excluded. To achieve comparability, nondiabetes patients aged below 30 years old at baseline were excluded. To minimize reverse causality, for both diabetes and nondiabetes patients, those who had less than 6 months of follow-up [23] were also excluded. Patients were followed up until a cancer diagnosis, death, or December 31, 2021, whichever occurred earlier. For patients who developed cancer at target organ sites during follow-up, those who received (i) a diagnosis of cancer at other nontarget cancer sites prior to the diagnosis of cancer at a target site or (ii) concurrent diagnoses of cancer at more than one target site were excluded. Patients who developed cancer at nontarget organ sites only were also excluded. Finally, 136,720 diabetes patients and 61,186 nondiabetes patients were selected.  Figure 1 shows the flow chart of patient selection.

### 2.3. Outcome Variables

The outcome of interest was the diagnosis of colorectal (ICD-10: C18-21), liver (ICD-10: C22), pancreatic (ICD-10: C25), bladder (ICD-10: C67), kidney (ICD-10: C64-66, C68), gastric (ICD-10: C16), and lung cancers (ICD-10: C33-34) during follow-up. These cancer sites are associated with diabetes [3] or obesity [24, 25] in the literature or of high cancer incidence [26].

### 2.4. Independent Variables

Independent variables included diabetes and metabolic factors (fasting glucose, lipids, and liver enzyme). Use of any antidiabetic drugs (metformin, sulfonylurea, insulin, dipeptidyl peptidase-4 inhibitors, acarbose, meglitinide, glitazone, sodium-glucose cotransporter-2 inhibitors, and glucagon-like peptide-1 receptor agonists) during the study period was treated as a proxy for a diagnosis of diabetes. Metabolic factors were laboratory measurements including ALT, fasting glucose, LDL cholesterol, HDL cholesterol, and triglycerides. Laboratory measurements were taken as the mean of at least two records closest to the baseline within 1 year. ALT was selected because it was the most commonly measured liver function test in the study cohort. Other covariates including demographics (sex and age), medication use, disease history, and lifestyle behavior (smoking) were added to control for potential confounding. Medication included antidiabetic drugs, aspirin, nonsteroidal anti-inflammatory drugs, anticoagulants, antiplatelets, statins, and antihypertensive drugs (angiotensin-converting enzyme inhibitors, angiotensin receptor blockers, alpha-blockers, beta-blockers, calcium channel blockers, and diuretics). Medication use was defined as whether patients had been prescribed a drug at baseline. Disease history included common comorbidities (ischemic heart disease, cerebrovascular disease, and heart failure) and risk factors for cancer (chronic hepatitis B/C [27], liver cirrhosis [27], fatty liver [27], chronic obstructive pulmonary disease [28], pneumonia [29], tuberculosis [28], haematuria [30], and cystitis) [30]. Disease diagnoses were determined from inpatient and outpatient diagnosis codes. The presence of fatty liver was determined from diagnosis codes and radiology reports (ultrasonography, computed tomography, and magnetic resonance imaging). Smoking status was extracted from clinical notes and defined as ever versus never smoker. Alcohol use was not available in this study.

### 2.5. Data Analysis

Baseline characteristics were presented as mean with standard deviation or median with interquartile range for continuous variables and as count with proportion for categorical variables. The associations between each studied factor and site-specific cancer risk were assessed using Cox proportional hazards regression, controlling for demographics, laboratory measurements, lifestyle behavior, disease history, and medication use. The estimates were reported as adjusted hazard ratio (aHR) with 95% confidence interval (CI). Model performance was evaluated using concordance (C-) index and integrated Brier score as metrics. Subgroup analyses were stratified by diabetes status. Sensitivity analyses were performed by (i) not excluding patients with a very short follow-up period; (ii) excluding patients who had a follow-up period of less than 1 year; and (iii) excluding patients who started to receive antidiabetic drugs within the first year of the prescription records (i.e., year 2000). Statistical significance was set at *p* < 0.05, two sided.

## 3. Results

A total of 197,906 patients were included. Among patients with diabetes (*n* = 136,720), the incidence rates of colorectal, liver, pancreatic, bladder, kidney, gastric, and lung cancers during follow-up (median: 5.58 years) were 1.84, 1.04, 0.53, 0.31, 0.23, 0.46, and 1.51 per 1000 person-years, respectively. The corresponding rates for patients without diabetes (*n* = 61,186; median of follow-up: 7.92 years) were 1.78, 0.61, 0.16, 0.30, 0.21, 0.43, and 1.72 per 1000 person-years.  Table 1 shows the baseline characteristics by diabetes status. Patients with diabetes tended to have higher levels of fasting glucose (8.11 vs. 5.40 mmol/L, *p* < 0.001), triglycerides (1.77 vs. 1.44 mmol/L, *p* < 0.001), and ALT (36.02 vs. 32.96 U/L, *p* < 0.001), but lower levels of LDL cholesterol (2.79 vs. 3.16 mmol/L, *p* < 0.001) and HDL cholesterol (1.24 vs. 1.36 mmol/L, *p* < 0.001) (Table 1). Baseline characteristics stratified by subsequent site-specific cancer development within diabetes and nondiabetes populations are shown in Tables S1a and S1b.

### 3.1. Diabetes and Site-Specific Cancer Risk

**Figure 2:**
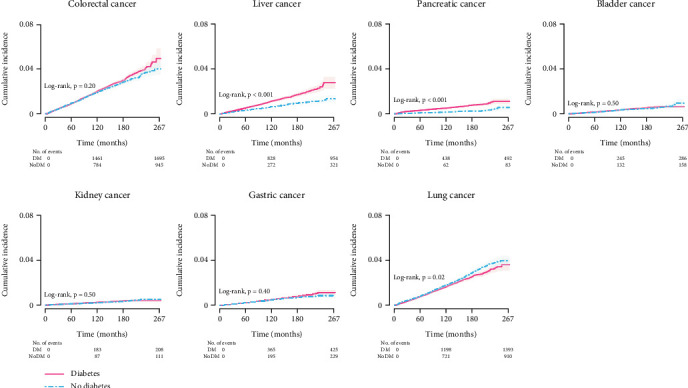
Cumulative site-specific cancer incidence by diabetes status.

Figure 2 shows the cumulative incidence curves for different site-specific cancers by diabetes status. The cumulative incidence curves appeared to be statistically different for liver, pancreatic, and lung cancers between patients with and without diabetes. Consistently, patients with diabetes had a higher risk of developing liver and pancreatic cancers (aHRs for liver: 1.39, 95% CI = 1.11–1.75; pancreas: 2.04, 95% CI = 1.40–2.96) during follow-up when compared to those without diabetes, after adjusting for age, sex, smoking, laboratory measurements, disease history, and medication use. The observed difference in lung cancer development by diabetes status disappeared after accounting for potential confounders (Table 2).

Overall, each 1 mmol/L increase in fasting glucose was associated with a 4% and 8% elevated risk of developing liver and pancreatic cancers, respectively (Table 2). Nevertheless, fasting glucose could be potentially positively linked to other digestive cancers (colon and rectum as well as stomach). Fasting glucose appeared to be positively associated with the risk of colorectal cancer across the diabetes (borderline significance, *p* = 0.07) and nondiabetes populations, but only positively linked to the risk of gastric cancer among the nondiabetes population (Tables 3 and 4).

### 3.2. Association of Liver Enzyme and Lipid Profile With Site-Specific Cancer Risk

Among patients with diabetes, ALT was positively associated with the risk of liver and pancreatic cancers, but negatively linked to the risk of colorectal cancer (Table 3). However, among patients without diabetes, ALT was only found to be positively associated with the risk of liver cancer (Table 4).

LDL cholesterol and triglycerides were inversely linked to the risk of liver and pancreatic cancers among patients with diabetes, but only negatively linked to the risk of liver cancer among patients without diabetes. On the other hand, HDL cholesterol was uniquely positively associated with the risk of liver cancer within the diabetes population, but inversely linked to the risk of gastric and kidney cancers among the diabetes and nondiabetes populations, respectively (Tables 3 and 4).

### 3.3. Model Performance

Across the overall, diabetes-only, and nondiabetes-only populations, the C-indexes of the fully adjusted models for different cancer sites ranged from 0.69 to 0.83 (Table 5). The corresponding integrated Brier scores ranged from 0.002 to 0.021 (Table S2). Overall, the model performance was acceptable [31].

### 3.4. Sensitivity Analyses

When patients with a very short follow-up period were not excluded, the results remained largely consistent, except for a positive association between diabetes and bladder cancer risk (Table S3). When patients with a follow-up period of less than 1 year or patients who initiated antidiabetic drugs within the first year of the prescription records were excluded, the results remained largely unchanged (Tables S4 and S5). There was insufficient evidence supporting the association between diabetes and bladder cancer after excluding patients with less than 6 or 12 months of follow-up (Table 2; Table S4).

## 4. Discussion

The current study utilized multiple linked datasets from electronic health records to systematically examine the associations between diabetes (primarily Type 2) and cancers across different organ sites among the Hong Kong population. Consistent with the literature, this study demonstrated a strong link between diabetes and cancers of the liver and pancreas. Nevertheless, there is also a potential association between elevated fasting glucose and other digestive cancers. While elevated ALT is known to be associated with an increased risk of liver cancer, findings of the study also suggest that ALT could be linked to pancreatic and colorectal cancers in opposite directions among patients with diabetes. Furthermore, the current study showed that lower levels of LDL cholesterol and triglycerides could be associated with an increased risk of liver and pancreatic cancers, while HDL cholesterol could be linked to some cancer sites in opposite directions.

In line with the literature, the present study demonstrated a strong association between diabetes and cancers of the liver and pancreas. A potential association between fasting glucose and other digestive cancers, including colon and rectum as well as stomach, was also found. However, diabetes was not shown to be associated with bladder, kidney, or lung cancers among the study cohort. Prior research has shown that diabetes is most strongly associated with liver and pancreatic cancers, but moderately with colorectal and bladder cancers [3, 4, 32–35]. However, the association between diabetes and gastric cancer remains controversial [5, 6]. Consistent with the findings of the present study, a recent study on diabetes and cancer [4] has also found that diabetes is most strongly linked to pancreatic and liver cancers (2.29- and 1.83-fold, respectively), after accounting for age and sex. The magnitudes of risk estimated in the present study were attenuated after additionally controlling for smoking, baseline laboratory measurements, disease history, and medication use.

Nevertheless, existing literature suggests the bidirectional nature of the association between diabetes and pancreatic cancer. Findings of the study suggested that the strength of the inverse associations between lipids and pancreatic cancer in diabetes became stronger when patients with a very short follow-up (less than 6 months) were not excluded. On the other hand, when the exclusion period extended from 6 months to 1 year, the observed inverse associations became less strong. Similarly, the association between diabetes and pancreatic cancer exhibited the same patterns. Prior research [36, 37] suggests that the recent onset of diabetes could be due to the manifestation of preexisting pancreatic cancer, while long-standing diabetes could be a risk factor for pancreatic cancer. It is estimated that diabetes is present in almost half of the pancreatic cancer cases [36], and up to 80% of the pancreatic cancer cases are either hyperglycemic or diabetic [36, 37]. On the other hand, 5%–10% of all patients with diabetes may have Type 3c diabetes mellitus (diabetes mellitus secondary to pancreatic diseases) [38]. Patients with pancreatic cancer-induced diabetes may exhibit a differential metabolic profile from those with primarily type 2 diabetes [39]. The diminishing strength in the observed associations with duration of follow-up may suggest the possibility of some potential remaining cases of pancreatic cancer-induced diabetes, despite the exclusion of early diagnosed cancer cases for up to 1 year. Further research is needed to explore the directionality of the relationship between diabetes and pancreatic cancer.

On the other hand, previous meta-analyses demonstrated that fasting glucose is independently positively associated with the risk of liver [10] and pancreatic [9] cancers. However, while a meta-analysis reported that fasting glucose and glycated hemoglobin were linked to an increased risk of colorectal cancer [40], a cohort study did not demonstrate any association between glycated hemoglobin and colorectal cancer [11]. Additionally, another recent meta-analysis showed that elevated fasting glucose was only associated with a borderline increased risk of gastric cancer when comparing patients with highest versus lowest categories [41]. Findings of the study support the associations between diabetes and cancers of the liver and pancreas and the potential links between elevated fasting glucose and other digestive cancers.

The exact mechanisms linking diabetes to cancers at different organ sites remain unclear. Several mechanisms linking diabetes to cancer include hyperinsulinemia, hyperglycemia, and chronic inflammation [3]. Previous research has shown that liver, pancreatic, colorectal, and gastric cancers are associated with obesity [24, 25]. Type 2 diabetes, the dominant form of diabetes, is due to the progressive loss of pancreatic *β*-cell insulin secretion and is characterized by insulin resistance and metabolic dysfunction [42]. Pathophysiologically, adipose tissue dysfunction in obesity may trigger insulin resistance and metabolic abnormalities (hyperinsulinemia and hyperglycemia) under a chronic state of low-grade inflammation [43, 44], promoting carcinogenesis [3]. Furthermore, in addition to the impaired insulin functioning at the pancreas [42] and ectopic fat accumulation at the liver [43], direct exposure to high levels of endogenous insulin at the pancreas and liver may increase the risk of pancreatic and liver cancers under diabetes condition [3]. Obesity-induced insulin resistance and physiological roles in insulin secretion and responses may partially explain the differential associations between diabetes and different cancer sites found in the present study.

Moreover, the current study revealed that lower circulating LDL cholesterol and triglycerides are potentially associated with an increased risk of liver cancer and additionally pancreatic cancer among patients with diabetes. While elevated LDL cholesterol and triglycerides are known to increase the risk of atherosclerotic cardiovascular diseases, the associations between lipids and cancer risk remain controversial [12, 13]. Growing evidence supports the potential inverse relationships between lipids and cancer risk [12, 13, 45]. A number of studies reported that lower cholesterol is associated with an increased risk of liver cancer [14, 15]. It has also been shown that a higher level of cholesterol may indicate preserved liver function and lower mortality [46]. Lower levels of lipids may signal a state of underlying illnesses [18]. In animal studies, accumulation of cholesterol in natural killer cells may activate effector functions against liver cancer cells [47]. Laboratory research further suggests that triglycerides synthesis could be downregulated in human liver cancer issues [48].

Paradoxically, poor health status (the presence of some chronic diseases such as chronic obstructive pulmonary disease [49] and chronic liver disease) [12] and cancer [12] itself are both associated with altered lipid metabolism. Prior research [18] has shown that the presence of illness, even a minor one, is associated with lower circulating lipids. Some evidence [50] also suggests that the inverse association may occur under an inflammatory state. The observed inverse associations of LDL cholesterol and triglycerides with cancer risk could be due to several hypothesized possibilities: (i) an altered lipid profile is associated with subsequent cancer risk; (ii) lower lipids as signals of underlying suboptimal health status; or (iii) lower lipids simply as manifestations of cancer symptoms.

On the other hand, the current study showed that HDL cholesterol exhibited a unique positive link to the risk of liver cancer among patients with diabetes. However, HDL cholesterol was negatively associated with the risk of gastric cancer among patients with diabetes and kidney cancer among patients without diabetes. While HDL cholesterol is known for its reverse cholesterol transport function against atherosclerosis [51], the association between HDL and cancer risk remains inconclusive [52]. The unique opposite relationships between HDL cholesterol and cancers at different sites by diabetes status could be partially explained by the heterogeneous nature of HDL particles, which vary in their functional and compositional properties [51]. Generally speaking, lower circulating lipids were found to be associated with an increased risk of several site-specific cancers in the current study. Two recent meta-analyses reported the inverse associations between circulating HDL cholesterol and cancers of the liver [45] and stomach [53] among the general population. This could be due to the cholesterol demand from tumors during carcinogenesis [13]. On the other hand, the unique positive association between circulating HDL cholesterol and liver cancer risk in diabetes observed in the current study could be due to recruitment of cholesterol from peripheral tissues to the liver to meet the demand of underlying liver tumor growth [13]. The relationship between HDL cholesterol and cancer risk in diabetes may also imply the role of altered lipid metabolism potentially due to ectopic fat deposition [12].

Furthermore, the present study found that while ALT is positively associated with the risk of liver cancer, ALT appears to be positively linked to pancreatic cancer but negatively linked to the risk of colorectal cancer among patients with diabetes. While ALT is a liver function marker commonly used to predict the risk of liver cancer [54], there is a paucity of studies on the associations between liver enzymes and cancers at other sites. A meta-analysis demonstrated mixed findings on the associations of ALT and overall cancer, with an inverse association in Europe and a positive association in Asia [17]. However, a recent prospective UK cohort study specific to colorectal cancer reported a negative association between ALT and colorectal cancer [55].

Since elevated ALT is an indicator of possible hepatic damage [16], the unique association between elevated ALT and increased pancreatic cancer risk among patients with diabetes could be due to the decline in pancreatic function in diabetes [42] leading to increased susceptibility to pancreatic cancer. On the other hand, one possible explanation for the inverse link between ALT and colorectal cancer among patients with diabetes is the disruption in gut–microbiota–liver axis (via portal vein) [56, 57], in particular proximal colon cancer [55, 58]. Diabetes is associated with liver diseases, which are in turn associated with altered gut microbiome, increased gut permeability, and intensified gut inflammation [56], potentially promoting carcinogenesis of the colon and rectum [57]. It is possible that the altered environment along the gut-liver axis [56] and potential underlying tumor growth at the colon and rectum may modify the supply and composition of gut-derived products to the liver, leading to changes in liver functioning. Furthermore, lower liver enzyme may also indicate potential underlying medical conditions. Specifically, lower ALT level is associated with an elevated risk of developing dementia [19] and considered as an indicator of frailty and sarcopenia in the elderly [59].

There are some potential public health and clinical implications of the current study. First, findings of the study may potentially provide evidence to inform cancer prevention strategies. Second, the associations between lipid profiles and site-specific cancer risk appear to be complex. While baseline lipid profiles were shown to be linked to the risk of liver cancer regardless of diabetes status, lipid profiles could be differentially associated with other cancer sites by diabetes status. Third, baseline ALT could also be differentially linked to the risk of other digestive cancers in opposite directions among patients with diabetes. Future research is warranted to test several hypotheses arisen from the current study: (i) lipids are generally inversely associated with the risk of cancer; (ii) lipids play a unique role in liver cancer development; (iii) ALT exhibits differential associations with the risk of other digestive cancers beyond the liver; and (iv) baseline profile of lipids and liver enzyme is differentially linked to the risk of cancers at different organ sites.

There are several strengths of the present study. First, this study covers health records of the public healthcare system from the entire population. The sample size is relatively large, hence enhancing the statistical power of the study. Second, the associations between diabetes and different site-specific cancers were systematically evaluated in a single study using homogenous approach, hence increasing the comparability of the estimated risk across cancer sites. Third, in addition to structured data, information from unstructured data has also been explored and incorporated into the set of covariates. Fourth, the use of multiple linked datasets may potentially improve confounding control. A previous review [8] has found that one-third of the studies may only be able to control for age and sex.

Several limitations may potentially exist in the present study. First, laboratory measurements (fasting glucose, lipids, and liver enzyme) were taken at baseline and may vary over time. However, the mean results of at least two records for each parameter were taken to reduce fluctuations between measurements. Second, the exclusion of patients with less than two records of each laboratory measurement within the first year of the index date may eliminate a proportion of patients without diabetes. However, this is to ensure that selected patients without diabetes achieve a similar level of interactions with the healthcare system as patients with diabetes, who are generally sicker and interact with the health system more frequently [23]. Third, since patients without diabetes are less likely to have complete laboratory records, together with the disproportionate distribution for certain cancer cases (pancreas), the number of some site-specific cancer cases among the nondiabetes population is relatively low. Fourth, the lower number of patients without diabetes may inherently limit the statistical power in detecting significant associations among the nondiabetes population. Nevertheless, only patients with complete laboratory records were selected. Fifth, the possibility of reverse causality between diabetes and cancer cannot be fully eliminated. Nevertheless, analyses were restricted to patients with at least 6 months of follow-up. Sensitivity analysis was also performed by excluding patients with a follow-up period of less than 1 year. However, there could be some pancreatic cancer-induced diabetes cases. Further studies with a longer follow-up focusing on long-standing diabetes (more than 5 years) [39] are warranted due to the bidirectional nature of diabetes and pancreatic cancer. Sixth, patients without diabetes selected in the present study may not be fully representative of patients without diabetes in the broader community. Seventh, to strengthen evidence for the relationship between diabetes and site-specific cancers, further studies with a longer length of follow-up are warranted. Eighth, information on some potential confounders such as diet patterns, alcohol use, and obesity indicators (for example, weight and waist circumference) was not available in this study. For example, obesity often coexists with diabetes and dyslipidemia and is closely linked to fatty liver disease [60], which is in turn associated with ALT level. Lean and obese individuals may exhibit differential patterns of metabolic profiles on different site-specific cancer risk. Ninth, cumulative medication dosage was not available in the present study. Lastly, histological subtypes or anatomic subsites of organ-specific cancers were not differentiated in the present study. Future research may explore the potential differential associations between diabetes and different histological subtypes/anatomic subsites of organ-specific cancers.

## 5. Conclusion

Diabetes is associated with an increased risk of liver and pancreatic cancers. Baseline lipids and liver enzymes may exhibit differential associations with the risk of cancers at different organ sites, and the patterns may differ by diabetes status. Findings of the study may potentially provide evidence to inform cancer prevention strategies for the diabetes population. Awareness of potential liver and pancreatic cancer symptoms should be raised in patients with diabetes to ensure early detection. The potential links between elevated fasting glucose and other digestive cancers, as well as the differential role of metabolic profiles in different site-specific cancers by diabetes status, may warrant further investigation.

## Figures and Tables

**Table 1:** Baseline characteristics of patients with and without diabetes.

**Characteristics**	**Diabetes**		**No diabetes**	
	**(** **n** = 136,720**)**		**(** **n** = 61,186**)**	
Demographics				
Male, *n* (%)	74,075	(54.18%)	34,686	(56.69%)
Age in year, mean ± SD	62.15	± 12.07	60.45	± 12.49
Follow-up time in month, median (IQR)	67	(34–112)	95	(51–137)
Laboratory measurements				
Fasting glucose in mmol/L, mean ± SD	8.11	± 2.38	5.40	± 0.66
Low-density lipoprotein cholesterol in mmol/L, mean ± SD	2.79	± 0.85	3.16	± 0.92
High-density lipoprotein cholesterol in mmol/L, mean ± SD	1.24	± 0.32	1.36	± 0.37
Triglycerides in mmol/L, mean ± SD	1.77	± 1.28	1.44	± 0.85
Alanine transaminase in U/L, mean ± SD	36.02	± 58.59	32.96	± 61.39
Lifestyle behavior				
Ever smoker, *n* (%)	27,465	(20.09%)	11,022	(18.01%)
Disease history				
Ischemic heart disease, *n* (%)	3614	(2.64%)	1375	(2.25%)
Cerebrovascular disease, *n* (%)	4734	(3.46%)	2151	(3.52%)
Heart failure, *n* (%)	5368	(3.93%)	996	(1.63%)
Chronic hepatitis B, *n* (%)	2142	(1.57%)	96	(0.16%)
Chronic hepatitis C, *n* (%)	286	(0.21%)	8	(0.01%)
Liver cirrhosis, *n* (%)	509	(0.37%)	17	(0.03%)
Fatty liver, *n* (%)	3114	(2.28%)	119	(0.19%)
Chronic obstructive pulmonary disease, *n* (%)	1129	(0.83%)	67	(0.11%)
Pneumonia, *n* (%)	346	(0.25%)	21	(0.03%)
Tuberculosis, *n* (%)	670	(0.49%)	54	(0.09%)
Haematuria, *n* (%)	4504	(3.29%)	530	(0.87%)
Cystitis, *n* (%)	5559	(4.07%)	794	(1.30%)
Medication use				
Antidiabetic drugs				
Metformin, *n* (%)	112,047	(81.95%)	0	(0%)
Sulfonylurea, *n* (%)	34,339	(25.12%)	0	(0%)
Insulin, *n* (%)	18,627	(13.62%)	0	(0%)
Dipeptidyl peptidase-4 inhibitors, *n* (%)	1666	(1.22%)	0	(0%)
Acarbose, *n* (%)	239	(0.17%)	0	(0%)
Meglitinide, *n* (%)	3	(0.00%)	0	(0%)
Glitazone, *n* (%)	657	(0.48%)	0	(0%)
Sodium-glucose cotransporter-2 inhibitors, *n* (%)	709	(0.52%)	0	(0%)
Glucagon-like peptide-1 receptor agonists, *n* (%)	58	(0.04%)	0	(0%)
Aspirin, *n* (%)	42,720	(31.25%)	18,902	(30.89%)
Nonsteroidal anti-inflammatory drugs, *n* (%)	82,166	(60.10%)	24,093	(39.38%)
Anticoagulants, *n* (%)	14,210	(10.39%)	4769	(7.79%)
Antiplatelets, *n* (%)	43,536	(31.84%)	19,245	(31.45%)
Statins, *n* (%)	71,056	(51.97%)	16,534	(27.02%)
Angiotensin-converting enzyme inhibitors, *n* (%)	51,639	(37.77%)	11,385	(18.61%)
Angiotensin receptor blockers, *n* (%)	15,996	(11.70%)	1841	(3.01%)
Alpha-blockers, *n* (%)	17,029	(12.46%)	3093	(5.06%)
Beta-blockers, *n* (%)	57,188	(41.83%)	16,516	(26.99%)
Calcium channel blockers, *n* (%)	72,441	(52.98%)	22,795	(37.26%)
Diuretics, *n* (%)	30,364	(22.21%)	5675	(9.27%)

Abbreviations: IQR, interquartile range; SD, standard deviation.

**Table 2:** Factors associated with site-specific cancer risk among the general population.

**Factor**	**Colon and rectum**	**Liver**	**Pancreas**	**Bladder**	**Kidney**	**Stomach**	**Lung**
	**aHR (95% CI)**	**aHR (95% CI)**	**aHR (95% CI)**	**aHR (95% CI)**	**aHR (95% CI)**	**aHR (95% CI)**	**aHR (95% CI)**
*Metabolic factors*							
Diabetes	1.02 (0.86–1.21)	**1.39 (1.11–1.75)**	**2.04 (1.40–2.96)**	1.42 (0.94–2.15)	1.03 (0.64–1.66)	1.06 (0.75–1.50)	1.08 (0.89–1.30)
Fasting glucose, every 1 mmol/L increase	**1.03 (1.01–1.05)**	**1.04 (1.01–1.07)**	**1.08 (1.04–1.12)**	0.97 (0.92–1.02)	0.97 (0.91–1.04)	1.03 (0.99–1.08)	0.99 (0.97–1.02)
Low-density lipoprotein cholesterol, every 1 mmol/L increase	1.00 (0.95–1.05)	**0.81 (0.75–0.87)**	**0.84 (0.75–0.94)**	1.03 (0.92–1.16)	1.01 (0.88–1.16)	1.01 (0.92–1.11)	1.00 (0.95–1.05)
High-density lipoprotein cholesterol, every 1 mmol/L increase	0.94 (0.83–1.07)	1.17 (0.98–1.38)	0.83 (0.63–1.10)	1.00 (0.74–1.35)	**0.65 (0.43–0.96)**	**0.64 (0.49–0.83)**	0.92 (0.81–1.06)
Triglycerides, every 1 mmol/L increase	1.02 (0.99–1.05)	**0.79 (0.72–0.85)**	**0.83 (0.74–0.93)**	1.04 (0.97–1.11)	0.90 (0.79–1.03)	0.94 (0.86–1.03)	0.98 (0.94–1.03)
Alanine transaminase, every 20 U/L increase	0.98 (0.96–1.00)	**1.02 (1.02–1.03)**	**1.02 (1.01–1.03)**	1.00 (0.97–1.04)	0.99 (0.94–1.04)	1.01 (0.99–1.03)	0.98 (0.96–1.00)
*Common risk factors*							
Male	**1.61 (1.46–1.76)**	**2.21 (1.91–2.55)**	**1.25 (1.02–1.52)**	**3.10 (2.37–4.05)**	**1.67 (1.27–2.21)**	**1.51 (1.25–1.82)**	**1.17 (1.05–1.30)**
Age, every 10-year increase	**1.69 (1.63–1.75)**	**1.51 (1.43–1.59)**	**1.69 (1.56–1.83)**	**1.98 (1.80–2.17)**	**1.13 (1.02–1.25)**	**1.79 (1.66–1.92)**	**1.76 (1.69–1.83)**
Ever smoker	**1.36 (1.24–1.49)**	**1.66 (1.47–1.88)**	**1.44 (1.17–1.76)**	**1.94 (1.58–2.38)**	**1.36 (1.05–1.76)**	**1.37 (1.14–1.64)**	**3.63 (3.30–4.00)**

*Note:* Models were adjusted for disease history (ischemic heart disease, cerebrovascular disease, heart failure, chronic hepatitis B/C, liver cirrhosis, fatty liver, chronic obstructive pulmonary disease, pneumonia, tuberculosis, haematuria, and cystitis) and medication use (antidiabetic drugs, aspirin, nonsteroidal anti-inflammatory drugs, anticoagulants, antiplatelets, statins, and antihypertensive drugs). Estimates in bold indicate statistical significance at an alpha level of 0.05.

Abbreviations: aHR, adjusted hazard ratio; CI, confidence interval.

**Table 3:** Factors associated with site-specific cancer risk among patients with diabetes.

**Factor**	**Colon and rectum**	**Liver**	**Pancreas**	**Bladder**	**Kidney**	**Stomach**	**Lung**
	**aHR (95% CI)**	**aHR (95% CI)**	**aHR (95% CI)**	**aHR (95% CI)**	**aHR (95% CI)**	**aHR (95% CI)**	**aHR (95% CI)**
Metabolic factors							
Fasting glucose, every 1 mmol/L increase	1.02 (1.00–1.04)	**1.03 (1.00–1.05)**	**1.08 (1.04–1.12)**	1.00 (1.00–1.00)	0.97 (0.91–1.04)	1.03 (0.98–1.07)	0.99 (0.97–1.01)
Low-density lipoprotein cholesterol, every 1 mmol/L increase	1.02 (0.96–1.08)	**0.81 (0.75–0.88)**	**0.82 (0.73–0.92)**	0.98 (0.92–1.03)	1.06 (0.89–1.25)	1.04 (0.92–1.17)	1.02 (0.96–1.09)
High-density lipoprotein cholesterol, every 1 mmol/L increase	1.01 (0.86–1.19)	**1.37 (1.12–1.66)**	0.77 (0.56–1.05)	1.00 (0.86–1.16)	0.75 (0.45–1.24)	**0.47 (0.33–0.68)**	0.92 (0.76–1.10)
Triglycerides, every 1 mmol/L increase	1.01 (0.98–1.05)	**0.82 (0.75–0.89)**	**0.82 (0.73–0.93)**	1.04 (0.71–1.54)	0.92 (0.80–1.07)	0.93 (0.83–1.03)	0.98 (0.93–1.03)
Alanine transaminase, every 20 U/L increase	**0.95 (0.92–0.99)**	**1.02 (1.02–1.03)**	**1.02 (1.01–1.03)**	1.00 (0.96–1.05)	1.00 (0.94–1.05)	1.01 (0.99–1.04)	0.98 (0.95–1.01)
Common risk factors							
Male	**1.68 (1.50–1.89)**	**2.10 (1.78–2.48)**	1.23 (0.99–1.53)	**2.99 (2.16–4.15)**	**1.64 (1.17–2.30)**	**1.42 (1.12–1.79)**	**1.22 (1.06–1.40)**
Age, every 10-year increase	**1.65 (1.58–1.73)**	**1.50 (1.40–1.59)**	**1.68 (1.54–1.83)**	**1.93 (1.72–2.18)**	1.04 (0.91–1.19)	**1.81 (1.65–1.99)**	**1.82 (1.72–1.92)**
Ever smoker	**1.39 (1.24–1.56)**	**1.62 (1.40–1.87)**	**1.45 (1.17–1.80)**	**1.87 (1.45–2.41)**	1.37 (0.99–1.90)	**1.35 (1.07–1.70)**	**3.53 (3.12–3.99)**

*Note:* Models were adjusted for disease history (ischemic heart disease, cerebrovascular disease, heart failure, chronic hepatitis B/C, liver cirrhosis, fatty liver, chronic obstructive pulmonary disease, pneumonia, tuberculosis, haematuria, and cystitis) and medication use (antidiabetic drugs, aspirin, nonsteroidal anti-inflammatory drugs, anticoagulants, antiplatelets, statins, and antihypertensive drugs). Estimates in bold indicate statistical significance at an alpha level of 0.05.

Abbreviations: aHR, adjusted hazard ratio; CI, confidence interval.

**Table 4:** Factors associated with site-specific cancer risk among patients without diabetes.

**Factor**	**Colon and rectum**	**Liver**	**Pancreas**	**Bladder**	**Kidney**	**Stomach**	**Lung**
	**aHR (95% CI)**	**aHR (95% CI)**	**aHR (95% CI)**	**aHR (95% CI)**	**aHR (95% CI)**	**aHR (95% CI)**	**aHR (95% CI)**
Metabolic factors							
Fasting glucose, every 1 mmol/L increase	**1.13 (1.04–1.23)**	**1.23 (1.10–1.38)**	1.05 (0.76–1.45)	0.78 (0.59–1.02)	0.85 (0.62–1.16)	**1.23 (1.06–1.43)**	1.07 (0.97–1.17)
Low-density lipoprotein cholesterol, every 1 mmol/L increase	0.98 (0.91–1.05)	**0.81 (0.71–0.93)**	0.97 (0.76–1.23)	1.08 (0.91–1.29)	0.92 (0.73–1.16)	0.98 (0.84–1.14)	0.97 (0.90–1.05)
High-density lipoprotein cholesterol, every 1 mmol/L increase	0.88 (0.72–1.07)	0.82 (0.58–1.15)	1.14 (0.66–1.98)	0.89 (0.55–1.46)	**0.50 (0.26–0.94)**	0.95 (0.64–1.42)	0.94 (0.77–1.15)
Triglycerides, every 1 mmol/L increase	1.06 (0.99–1.13)	**0.68 (0.55–0.84)**	0.87 (0.63–1.20)	0.99 (0.79–1.24)	0.84 (0.63–1.13)	0.97 (0.81–1.17)	1.00 (0.92–1.10)
Alanine transaminase, every 20 U/L increase	1.00 (0.98–1.02)	**1.03 (1.02–1.04)**	1.01 (0.96–1.07)	1.01 (0.96–1.05)	0.98 (0.88–1.09)	0.99 (0.93–1.06)	0.98 (0.95–1.02)
Common risk factors							
Male	**1.47 (1.26–1.71)**	**2.42 (1.81–3.26)**	1.40 (0.90–2.18)	**3.43 (2.15–5.47)**	**1.74 (1.08–2.81)**	**1.69 (1.23–2.32)**	1.10 (0.92–1.30)
Age, every 10-year increase	**1.73 (1.63–1.83)**	**1.55 (1.40–1.71)**	**1.74 (1.44–2.09)**	**2.05 (1.77–2.39)**	**1.27 (1.07–1.50)**	**1.73 (1.54–1.95)**	**1.67 (1.58–1.78)**
Ever smoker	**1.31 (1.12–1.53)**	**1.85 (1.45–2.37)**	1.31 (0.81–2.14)	**2.07 (1.48–2.90)**	1.32 (0.85–2.04)	**1.40 (1.03–1.90)**	**3.85 (3.31–4.48)**

*Note:* Models were adjusted for disease history (ischemic heart disease, cerebrovascular disease, heart failure, chronic hepatitis B/C, liver cirrhosis, fatty liver, chronic obstructive pulmonary disease, pneumonia, tuberculosis, haematuria, and cystitis) and medication use (aspirin, nonsteroidal anti-inflammatory drugs, anticoagulants, antiplatelets, statins, and antihypertensive drugs). Estimates in bold indicate statistical significance at an alpha level of 0.05.

Abbreviations: aHR, adjusted hazard ratio; CI, confidence interval.

**Table 5:** Model performance across different site-specific cancers by diabetes status and cancer site using concordance index as a metric.

**Overall**			**Diabetes**			**No diabetes**		
**Rank**	**Cancer site**	**Concordance index (95% CI)**	**Rank**	**Cancer site**	**Concordance index (95% CI)**	**Rank**	**Cancer site**	**Concordance index (95% CI)**
1	Bladder	0.803 (0.783–0.823)	1	Bladder	0.791 (0.766–0.816)	1	Bladder	0.831 (0.804–0.858)
2	Liver	0.784 (0.770–0.798)	2	Liver	0.785 (0.769–0.801)	2	Liver	0.785 (0.769–0.801)
3	Pancreas	0.756 (0.736–0.776)	3	Lung	0.755 (0.741–0.769)	3	Lung	0.757 (0.741–0.773)
4	Lung	0.755 (0.745–0.765)	4	Pancreas	0.723 (0.699–0.747)	4	Stomach	0.750 (0.721–0.779)
5	Stomach	0.719 (0.699–0.739)	5	Stomach	0.715 (0.690–0.740)	5	Pancreas	0.748 (0.697–0.799)
6	Colon and rectum	0.700 (0.690–0.710)	6	Kidney	0.706 (0.667–0.745)	6	Colon and rectum	0.720 (0.704–0.736)
6	Kidney	0.700 (0.669–0.731)	7	Colon and rectum	0.693 (0.679–0.707)	7	Kidney	0.709 (0.654–0.764)

Abbreviation: CI, confidence interval.

## Data Availability

Data is not available for sharing due to access restriction.

## Supporting Information

Supporting Information Additional supporting information can be found online in the Supporting Information section. Table S1a: Baseline characteristics of patients with diabetes by site-specific cancer incidence. Table S1b: Baseline characteristics of patients without diabetes by site-specific cancer incidence. Table S2: Model performance across different site-specific cancers by diabetes status and cancer site using integrated Brier score as a metric. Table S3: Sensitivity analysis—factors associated with site-specific cancer risk without excluding patients with a very short follow-up period. Table S4: Sensitivity analysis—factors associated with site-specific cancer risk after excluding patients with a follow-up period of less than 1 year. Table S5: Sensitivity analysis—association between diabetes and site-specific cancer risk after excluding a diagnosis of diabetes within the first year of the prescription records.
