# Supplementary material for: Association Between Diabetes and Site-Specific Cancer Risk: A Population-Based Cohort Study on the Differential Role of Metabolic Profiles
Source: J Diabetes Res. 2025 Aug 11;2025:1271189. doi: 10.1155/jdr/1271189 (PMC12360884; doi:10.1155/jdr/1271189)
Supplement: Supporting Information — Additional supporting information can be found online in the Supporting Information section. Table S1a: Baseline characteristics of patients with diabetes by site-specific cancer incidence. Table S1b: Baseline characteristics of patients without diabetes by site-specific cancer incidence. Table S2: Model performance across different site-specific cancers by diabetes status and cancer site using integrated Brier score as a metric. Table S3: Sensitivity analysis—factors associated with site-specific cancer risk without excluding patients with a very short follow-up period. Table S4: Sensitivity analysis—factors associated with site-specific cancer risk after excluding patients with a follow-up period of less than 1 year. Table S5: Sensitivity analysis—association between diabetes and site-specific cancer risk after excluding a diagnosis of diabetes within the first year of the prescription records. [file 1271189.f1.docx]

Table S1a. Baseline characteristics of patients with diabetes by site-specific cancer incidence.

|  | **Colon & Rectum** | | **Liver** | | **Pancreas** | | **Bladder** | | **Kidney** | | **Stomach** | | **Lung** | | **No Cancer** | |
| --- | --- | --- | --- | --- | --- | --- | --- | --- | --- | --- | --- | --- | --- | --- | --- | --- |
| **Characteristics** | **(n=1,695)** | | **(n=954)** | | **(n=492)** | | **(n=286)** | | **(n=208)** | | **(n=425)** | | **(n=1,393)** | | **(n=131,267)** | |
| Demographics |  |  |  |  |  |  |  |  |  |  |  |  |  |  |  |  |
| Male, n (%) | 1,094 | (64.50%) | 699 | (73.27%) | 295 | (59.96%) | 226 | (79.02%) | 139 | (66.83%) | 269 | (63.29%) | 936 | (67.19%) | 70,417 | (53.64%) |
| Age in year, mean±SD | 66.26 | ±10.33 | 64.03 | ±9.71 | 66.80 | ±10.80 | 68.30 | ±9.97 | 62.51 | ±11.63 | 67.12 | ±10.56 | 67.50 | ±9.77 | 61.98 | ±12.11 |
| Follow-up time in month, median (IQR) | 55 | (25.5-93) | 52 | (24-93) | 30 | (14-74) | 53 | (29-94) | 44.5 | (18-82.5) | 54 | (25-91.5) | 54 | (27-91) | 68 | (35-112) |
| Laboratory measurements |  |  |  |  |  |  |  |  |  |  |  |  |  |  |  |  |
| Fasting glucose in mmol/L, mean±SD | 8.29 | ±2.35 | 8.41 | ±2.51 | 8.47 | ±2.70 | 8.03 | ±2.34 | 7.95 | ±2.49 | 8.27 | ±2.60 | 8.05 | ±2.32 | 8.11 | ±2.38 |
| Low-density lipoprotein cholesterol in mmol/L, mean±SD | 2.89 | ±0.83 | 2.76 | ±0.85 | 2.73 | ±0.88 | 2.83 | ±0.77 | 2.86 | ±0.81 | 2.89 | ±0.82 | 2.87 | ±0.85 | 2.79 | ±0.85 |
| High-density lipoprotein cholesterol in mmol/L, mean±SD | 1.23 | ±0.31 | 1.27 | ±0.37 | 1.24 | ±0.31 | 1.21 | ±0.33 | 1.20 | ±0.04 | 1.18 | ±0.30 | 1.22 | ±0.31 | 1.24 | ±0.32 |
| Triglycerides in mmol/L, mean±SD | 1.76 | ±1.96 | 1.50 | ±1.13 | 1.59 | ±0.84 | 1.75 | ±1.08 | 1.75 | ±0.97 | 1.72 | ±1.03 | 1.70 | ±1.05 | 1.77 | ±1.27 |
| Alanine transaminase in U/L, mean±SD | 30.19 | ±31.17 | 59.32 | ±71.31 | 42.37 | ±75.13 | 32.74 | ±29.72 | 35.2 | ±47.22 | 35.88 | ±50.32 | 31.02 | ±40.93 | 35.97 | ±58.90 |
| Lifestyle behavior |  |  |  |  |  |  |  |  |  |  |  |  |  |  |  |  |
| Ever smoker, n (%) | 521 | (30.72%) | 349 | (36.58%) | 138 | (28.05%) | 126 | (44.06%) | 64 | (30.77%) | 127 | (29.88%) | 699 | (50.18%) | 25,441 | (19.38%) |
| Disease history |  |  |  |  |  |  |  |  |  |  |  |  |  |  |  |  |
| Ischemic heart disease, n (%) | 48 | (2.83%) | 22 | (2.31%) | 6 | (1.22%) | 14 | (4.90%) | 2 | (0.96%) | 11 | (2.59%) | 56 | (4.02%) | 3,455 | (2.63%) |
| Cerebrovascular disease, n (%) | 68 | (4.01%) | 37 | (3.88%) | 12 | (2.44%) | 18 | (6.29%) | 8 | (3.85%) | 9 | (2.12%) | 53 | (3.80%) | 4,529 | (3.45%) |
| Heart failure, n (%) | 66 | (3.89%) | 36 | (3.77%) | 16 | (3.25%) | 10 | (3.50%) | 8 | (3.85%) | 22 | (5.18%) | 46 | (3.30%) | 5,164 | (3.93%) |
| Chronic hepatitis B, n (%) | 17 | (1.00%) | 116 | (12.16%) | 10 | (2.03%) | 1 | (0.35%) | 4 | (1.92%) | 5 | (1.18%) | 14 | (1.01%) | 1,975 | (1.50%) |
| Chronic hepatitis C, n (%) | 1 | (0.06%) | 29 | (3.04%) | 3 | (0.61%) | 0 | (0%) | 0 | (0%) | 0 | (0%) | 4 | (0.29%) | 249 | (0.19%) |
| Liver cirrhosis, n (%) | 6 | (0.35%) | 64 | (6.71%) | 5 | (1.02%) | 0 | (0%) | 0 | (0%) | 2 | (0.47%) | 6 | (0.43%) | 426 | (0.32%) |
| Fatty liver, n (%) | 13 | (0.77%) | 27 | (2.83%) | 11 | (2.24%) | 2 | (0.70%) | 4 | (1.92%) | 11 | (2.59%) | 18 | (1.29%) | 3,028 | (2.31%) |
| Chronic obstructive pulmonary disease, n (%) | 11 | (0.65%) | 7 | (0.73%) | 3 | (0.61%) | 4 | (1.40%) | 1 | (0.48%) | 2 | (0.47%) | 17 | (1.22%) | 1,084 | (0.83%) |
| Pneumonia, n (%) | 3 | (0.18%) | 4 | (0.42%) | 0 | (0%) | 3 | (1.05%) | 0 | (0%) | 1 | (0.24%) | 5 | (0.36%) | 330 | (0.25%) |
| Tuberculosis, n (%) | 7 | (0.41%) | 9 | (0.94%) | 1 | (0.20%) | 1 | (0.35%) | 1 | (0.48%) | 2 | (0.47%) | 24 | (1.72%) | 625 | (0.48%) |
| Haematuria, n (%) | 38 | (2.24%) | 32 | (3.35%) | 19 | (3.86%) | 17 | (5.94%) | 11 | (5.29%) | 8 | (1.88%) | 34 | (2.44%) | 4,345 | (3.31%) |
| Cystitis, n (%) | 32 | (1.89%) | 17 | (1.78%) | 16 | (3.25%) | 9 | (3.15%) | 12 | (5.77%) | 13 | (3.06%) | 36 | (2.58%) | 5,424 | (4.13%) |
| Medication use |  |  |  |  |  |  |  |  |  |  |  |  |  |  |  |  |
| Anti-diabetic drugs |  |  |  |  |  |  |  |  |  |  |  |  |  |  |  |  |
| Metformin, n (%) | 1,266 | (74.65%) | 687 | (72.01%) | 398 | (80.89%) | 194 | (67.83%) | 151 | (72.60%) | 320 | (75.29%) | 1,037 | (74.44%) | 107,994 | (82.27%) |
| Sulfonylurea, n (%) | 612 | (36.08%) | 391 | (40.99%) | 156 | (31.71%) | 118 | (41.26%) | 70 | (33.65%) | 156 | (36.71%) | 451 | (32.38%) | 32,385 | (24.67%) |
| Insulin, n (%) | 196 | (11.56%) | 127 | (13.31%) | 42 | (8.54%) | 29 | (10.14%) | 25 | (12.02%) | 30 | (7.06%) | 159 | (11.41%) | 18,019 | (13.73%) |
| Dipeptidyl peptidase-4 inhibitors, n (%) | 10 | (0.59%) | 9 | (0.94%) | 6 | (1.22%) | 1 | (0.35%) | 8 | (3.85%) | 4 | (0.94%) | 6 | (0.43%) | 1,622 | (1.24%) |
| Acarbose, n (%) | 3 | (0.18%) | 2 | (0.21%) | 1 | (0.20%) | 1 | (0.35%) | 0 | (0%) | 0 | (0%) | 3 | (0.22%) | 229 | (0.17%) |
| Meglitinide, n (%) | 0 | (0%) | 0 | (0%) | 0 | (0%) | 0 | (0%) | 0 | (0%) | 0 | (0%) | 1 | (0.07%) | 2 | (0.00%) |
| Glitazone, n (%) | 2 | (0.12%) | 2 | (0.21%) | 6 | (1.22%) | 1 | (0.35%) | 0 | (0%) | 3 | (0.71%) | 2 | (0.14%) | 641 | (0.49%) |
| Sodium-glucose cotransporter-2 inhibitors, n (%) | 0 | (0%) | 0 | (0%) | 1 | (0.20%) | 0 | (0%) | 0 | (0%) | 0 | (0%) | 1 | (0.07%) | 707 | (0.54%) |
| Glucagon-like peptide-1 receptor agonists, n (%) | 0 | (0%) | 0 | (0%) | 0 | (0%) | 0 | (0%) | 0 | (0%) | 0 | (0%) | 1 | (0.07%) | 57 | (0.04%) |
| Aspirin, n (%) | 527 | (31.07%) | 228 | (23.90%) | 137 | (27.85%) | 119 | (41.61%) | 75 | (36.06%) | 137 | (32.24%) | 508 | (36.47%) | 40,989 | (31.23%) |
| Non-steroidal anti-inflammatory drugs, n (%) | 797 | (46.99%) | 439 | (46.02%) | 278 | (56.50%) | 130 | (45.45%) | 109 | (52.40%) | 210 | (49.41%) | 708 | (50.83%) | 79,495 | (60.56%) |
| Anti-coagulants, n (%) | 166 | (9.79%) | 69 | (7.23%) | 38 | (7.72%) | 30 | (10.49%) | 20 | (9.62%) | 40 | (9.41%) | 148 | (10.62%) | 13,699 | (10.44%) |
| Anti-platelets, n (%) | 535 | (31.54%) | 231 | (24.21%) | 138 | (28.05%) | 120 | (41.96%) | 75 | (36.06%) | 139 | (32.71%) | 513 | (36.83%) | 41,785 | (31.83%) |
| Statins, n (%) | 772 | (45.52%) | 266 | (27.88%) | 244 | (49.59%) | 142 | (49.65%) | 118 | (56.73%) | 193 | (45.41%) | 689 | (49.46%) | 68,632 | (52.28%) |
| Angiotensin-converting enzyme inhibitors, n (%) | 655 | (38.62%) | 310 | (32.49%) | 184 | (37.40%) | 119 | (41.61%) | 106 | (50.96%) | 178 | (41.88%) | 545 | (39.12%) | 49,542 | (37.74%) |
| Angiotensin receptor blockers, n (%) | 134 | (7.90%) | 46 | (4.82%) | 40 | (8.13%) | 18 | (6.29%) | 24 | (11.54%) | 30 | (7.06%) | 79 | (5.67%) | 15,626 | (11.90%) |
| Alpha-blockers, n (%) | 210 | (12.38%) | 124 | (13.00%) | 80 | (16.26%) | 53 | (18.53%) | 37 | (17.79%) | 63 | (14.82%) | 199 | (14.29%) | 16,263 | (12.39%) |
| Beta-blockers, n (%) | 721 | (42.51%) | 404 | (42.35%) | 199 | (40.45%) | 121 | (42.31%) | 109 | (52.40%) | 168 | (39.53%) | 598 | (42.93%) | 54,868 | (41.80%) |
| Calcium channel blockers, n (%) | 882 | (52.00%) | 436 | (45.70%) | 272 | (55.28%) | 145 | (50.70%) | 134 | (64.42%) | 227 | (53.41%) | 722 | (51.83%) | 69,623 | (53.04%) |
| Diuretics, n (%) | 399 | (23.53%) | 231 | (24.21%) | 122 | (24.80%) | 64 | (22.38%) | 59 | (28.37%) | 99 | (23.29%) | 311 | (22.33%) | 29,079 | (22.15%) |

IQR, interquartile range; SD, standard deviation.

Table S1b. Baseline characteristics of patients without diabetes by site-specific cancer incidence.

|  | **Colon & Rectum** | | **Liver** | | **Pancreas** | | **Bladder** | | **Kidney** | | **Stomach** | | **Lung** | | **No Cancer** | |
| --- | --- | --- | --- | --- | --- | --- | --- | --- | --- | --- | --- | --- | --- | --- | --- | --- |
| **Characteristics** | **(n=945)** | | **(n=321)** | | **(n=83)** | | **(n=158)** | | **(n=111)** | | **(n=229)** | | **(n=910)** | | **(n=58,429)** | |
| Demographics |  |  |  |  |  |  |  |  |  |  |  |  |  |  |  |  |
| Male, n (%) | 607 | (64.23%) | 247 | (76.95%) | 50 | (60.24%) | 131 | (82.91%) | 81 | (72.97%) | 154 | (67.25%) | 620 | (68.13%) | 32,796 | (56.13%) |
| Age in year, mean±SD | 67.22 | ±10.52 | 65.42 | ±10.81 | 66.66 | ±10.96 | 68.90 | ±9.56 | 63.32 | ±11.07 | 67.38 | ±10.28 | 66.88 | ±9.54 | 60.15 | ±12.51 |
| Follow-up time in month, median (IQR) | 60 | (31-101) | 53 | (25-98) | 61 | (14-74) | 72 | (29-94) | 55 | (18-82.5) | 57 | (25-91.5) | 66.5 | (27-91) | 97 | (35-112) |
| Laboratory measurements |  |  |  |  |  |  |  |  |  |  |  |  |  |  |  |  |
| Fasting glucose in mmol/L, mean±SD | 5.48 | ±0.76 | 5.55 | ±0.89 | 5.41 | ±0.75 | 5.34 | ±0.58 | 5.35 | ±0.67 | 5.53 | ±0.73 | 5.44 | ±0.68 | 5.40 | ±0.66 |
| Low-density lipoprotein cholesterol in mmol/L, mean±SD | 3.18 | ±0.82 | 3.01 | ±1.15 | 3.17 | ±0.99 | 3.21 | ±0.97 | 3.10 | ±0.76 | 3.17 | ±0.86 | 3.17 | ±0.90 | 3.16 | ±0.92 |
| High-density lipoprotein cholesterol in mmol/L, mean±SD | 1.33 | ±0.36 | 1.33 | ±0.38 | 1.40 | ±0.38 | 1.31 | ±0.34 | 1.26 | ±0.35 | 1.34 | ±0.36 | 1.34 | ±0.37 | 1.36 | ±0.38 |
| Triglycerides in mmol/L, mean±SD | 1.47 | ±0.92 | 1.27 | ±0.58 | 1.34 | ±0.58 | 1.39 | ±0.60 | 1.42 | ±0.61 | 1.40 | ±0.62 | 1.43 | ±0.68 | 1.45 | ±0.86 |
| Alanine transaminase in U/L, mean±SD | 30.62 | ±82.79 | 57.75 | ±67.19 | 32.71 | ±32.72 | 30.65 | ±24.47 | 29.86 | ±23.68 | 29.80 | ±29.33 | 28.84 | ±42.69 | 32.95 | ±61.41 |
| Lifestyle behavior |  |  |  |  |  |  |  |  |  |  |  |  |  |  |  |  |
| Ever smoker, n (%) | 262 | (27.72%) | 118 | (36.76%) | 22 | (26.51%) | 72 | (45.57%) | 34 | (30.63%) | 68 | (29.69%) | 459 | (50.44%) | 9,987 | (17.09%) |
| Disease history |  |  |  |  |  |  |  |  |  |  |  |  |  |  |  |  |
| Ischemic heart disease, n (%) | 23 | (2.43%) | 5 | (1.56%) | 1 | (1.20%) | 4 | (2.53%) | 2 | (1.80%) | 2 | (0.87%) | 28 | (3.08%) | 1,310 | (2.24%) |
| Cerebrovascular disease, n (%) | 31 | (3.28%) | 17 | (5.30%) | 0 | (0%) | 11 | (6.96%) | 8 | (7.21%) | 10 | (4.37%) | 41 | (4.51%) | 2,033 | (3.48%) |
| Heart failure, n (%) | 24 | (2.54%) | 7 | (2.18%) | 0 | (0%) | 3 | (1.90%) | 4 | (3.60%) | 6 | (2.62%) | 15 | (1.65%) | 937 | (1.60%) |
| Chronic hepatitis B, n (%) | 0 | (0%) | 10 | (3.12%) | 0 | (0%) | 0 | (0%) | 0 | (0%) | 0 | (0%) | 1 | (0.11%) | 85 | (0.15%) |
| Chronic hepatitis C, n (%) | 0 | (0%) | 0 | (0%) | 1 | (1.20%) | 0 | (0%) | 0 | (0%) | 0 | (0%) | 0 | (0%) | 7 | (0.01%) |
| Liver cirrhosis, n (%) | 1 | (0.11%) | 2 | (0.62%) | 0 | (0%) | 0 | (0%) | 0 | (0%) | 0 | (0%) | 1 | (0.11%) | 13 | (0.02%) |
| Fatty liver, n (%) | 0 | (0%) | 1 | (0.31%) | 0 | (0%) | 0 | (0%) | 0 | (0%) | 0 | (0%) | 0 | (0%) | 118 | (0.20%) |
| Chronic obstructive pulmonary disease, n (%) | 0 | (0%) | 0 | (0%) | 0 | (0%) | 0 | (0%) | 0 | (0%) | 1 | (0.44%) | 3 | (0.33%) | 63 | (0.11%) |
| Pneumonia, n (%) | 1 | (0.11%) | 0 | (0%) | 0 | (0%) | 0 | (0%) | 0 | (0%) | 0 | (0%) | 1 | (0.11%) | 19 | (0.03%) |
| Tuberculosis, n (%) | 0 | (0%) | 0 | (0%) | 0 | (0%) | 1 | (0.63%) | 1 | (0.90%) | 0 | (0%) | 1 | (0.11%) | 51 | (0.09%) |
| Haematuria, n (%) | 4 | (0.42%) | 2 | (0.62%) | 0 | (0%) | 5 | (3.16%) | 2 | (1.80%) | 0 | (0%) | 7 | (0.77%) | 510 | (0.87%) |
| Cystitis, n (%) | 7 | (0.74%) | 5 | (1.56%) | 0 | (0%) | 3 | (1.90%) | 0 | (0%) | 3 | (1.31%) | 7 | (0.77%) | 769 | (1.32%) |
| Medication use |  |  |  |  |  |  |  |  |  |  |  |  |  |  |  |  |
| Aspirin, n (%) | 312 | (33.02%) | 90 | (28.04%) | 25 | (30.12%) | 60 | (37.97%) | 47 | (42.34%) | 77 | (33.62%) | 357 | (39.23%) | 17,934 | (30.69%) |
| Non-steroidal anti-inflammatory drugs, n (%) | 281 | (29.74%) | 117 | (36.45%) | 33 | (39.76%) | 47 | (29.75%) | 41 | (36.94%) | 75 | (32.75%) | 320 | (35.16%) | 23,179 | (39.67%) |
| Anti-coagulants, n (%) | 81 | (8.57%) | 21 | (6.54%) | 8 | (9.64%) | 16 | (10.13%) | 8 | (7.21%) | 22 | (9.61%) | 80 | (8.79%) | 4,533 | (7.76%) |
| Anti-platelets, n (%) | 322 | (34.07%) | 91 | (28.35%) | 26 | (31.33%) | 62 | (39.24%) | 47 | (42.34%) | 79 | (34.50%) | 362 | (39.78%) | 18,256 | (31.24%) |
| Statins, n (%) | 243 | (25.71%) | 59 | (18.38%) | 23 | (27.71%) | 43 | (27.22%) | 35 | (31.53%) | 55 | (24.02%) | 259 | (28.46%) | 15,817 | (27.07%) |
| Angiotensin-converting enzyme inhibitors, n (%) | 216 | (22.86%) | 64 | (19.94%) | 12 | (14.46%) | 35 | (22.15%) | 33 | (29.73%) | 39 | (17.03%) | 189 | (20.77%) | 10,797 | (18.48%) |
| Angiotensin receptor blockers, n (%) | 21 | (2.22%) | 13 | (4.05%) | 3 | (3.61%) | 2 | (1.27%) | 1 | (0.90%) | 1 | (0.44%) | 22 | (2.42%) | 1,778 | (3.04%) |
| Alpha-blockers, n (%) | 75 | (7.94%) | 24 | (7.48%) | 7 | (8.43%) | 26 | (16.46%) | 11 | (9.91%) | 19 | (8.30%) | 69 | (7.58%) | 2,862 | (4.90%) |
| Beta-blockers, n (%) | 328 | (34.71%) | 101 | (31.46%) | 25 | (30.12%) | 44 | (27.85%) | 47 | (42.34%) | 75 | (32.75%) | 276 | (30.33%) | 15,620 | (26.73%) |
| Calcium channel blockers, n (%) | 385 | (40.74%) | 146 | (45.48%) | 31 | (37.35%) | 57 | (36.08%) | 49 | (44.14%) | 99 | (43.23%) | 334 | (36.70%) | 21,694 | (37.13%) |
| Diuretics, n (%) | 123 | (13.02%) | 37 | (11.53%) | 11 | (13.25%) | 23 | (14.56%) | 14 | (12.61%) | 22 | (9.61%) | 113 | (12.42%) | 5,332 | (9.13%) |

IQR, interquartile range; SD, standard deviation.

Table S2. Model performance across different site-specific cancers by diabetes status and cancer site using integrated Brier score as metric.

|  | **Overall** |  | **Diabetes** |  | **No Diabetes** |
| --- | --- | --- | --- | --- | --- |
| **Cancer site** | **Integrated Brier Score** |  | **Integrated Brier Score** |  | **Integrated Brier Score** |
| Colon & Rectum | 0.020 |  | 0.021 |  | 0.019 |
| Liver | 0.010 |  | 0.012 |  | 0.007 |
| Pancreas | 0.004 |  | 0.006 |  | 0.002 |
| Bladder | 0.004 |  | 0.004 |  | 0.004 |
| Kidney | 0.003 |  | 0.003 |  | 0.003 |
| Stomach | 0.005 |  | 0.006 |  | 0.005 |
| Lung | 0.018 |  | 0.017 |  | 0.019 |

Table S3. Sensitivity analysis - Factors associated with site-specific cancer risk without excluding patients with a very short follow-up period.

i) Overall

|  | **Overall** | | | | | | |
| --- | --- | --- | --- | --- | --- | --- | --- |
|  | **Colon & Rectum** | **Liver** | **Pancreas** | **Bladder** | **Kidney** | **Stomach** | **Lung** |
| **Factor** | **aHR (95%CI)** | **aHR (95%CI)** | **aHR (95%CI)** | **aHR (95%CI)** | **aHR (95%CI)** | **aHR (95%CI)** | **aHR (95%CI)** |
| Metabolic factors |  |  |  |  |  |  |  |
| Diabetes | 1.12 (0.97-1.30) | **1.46 (1.18-1.81)** | **2.39 (1.72-3.32)** | **1.55 (1.05-2.28)** | 1.06 (0.68-1.67) | 1.13 (0.81-1.56) | 0.99 (0.83-1.18) |
| Fasting glucose, every 1 mmol/L increase | **1.02 (1.01-1.04)** | **1.03 (1.01-1.06)** | **1.10 (1.07-1.13)** | 0.98 (0.93-1.03) | 0.96 (0.89-1.03) | 1.03 (0.99-1.07) | 1.00 (0.98-1.02) |
| Low-density lipoprotein cholesterol, every 1 mmol/L increase | **0.95 (0.91-0.99)** | **0.80 (0.73-0.89)** | **0.83 (0.75-0.91)** | 1.02 (0.92-1.14) | 1.02 (0.90-1.16) | 0.95 (0.87-1.04) | 0.99 (0.95-1.04) |
| High-density lipoprotein cholesterol, every 1 mmol/L increase | 0.89 (0.80-1.01) | 1.07 (0.91-1.26) | **0.73 (0.56-0.94)** | 1.01 (0.76-1.33) | **0.60 (0.41-0.88)** | **0.57 (0.44-0.74)** | 0.88 (0.78-1.00) |
| Triglycerides, every 1 mmol/L increase | 1.00 (0.96-1.04) | **0.77 (0.71-0.83)** | **0.77 (0.69-0.85)** | 1.04 (0.98-1.11) | 0.91 (0.80-1.03) | **0.91 (0.82-0.99)** | 0.97 (0.93-1.02) |
| Alanine transaminase, every 20 U/L increase | **0.96 (0.94-0.98)** | **1.02 (1.02-1.03)** | **1.02 (1.02-1.03)** | 1.01 (0.98-1.03) | 1.00 (0.96-1.04) | 1.01 (0.98-1.03) | 0.98 (0.95-1.00) |
| Common risk factors |  |  |  |  |  |  |  |
| Male | **1.58 (1.45-1.73)** | **2.26 (1.97-2.59)** | 1.11 (0.93-1.33) | **2.98 (2.31-3.83)** | **1.62 (1.25-2.11)** | **1.50 (1.25-1.80)** | **1.18 (1.07-1.31)** |
| Age, every 10-year increase | **1.68 (1.63-1.74)** | **1.51 (1.43-1.58)** | **1.67 (1.55-1.79)** | **1.98 (1.81-2.16)** | **1.12 (1.01-1.23)** | **1.80 (1.68-1.93)** | **1.73 (1.67-1.80)** |
| Ever smoker | **1.38 (1.27-1.51)** | **1.63 (1.45-1.83)** | **1.34 (1.11-1.62)** | **2.05 (1.69-2.48)** | **1.39 (1.09-1.78)** | **1.38 (1.16-1.64)** | **3.51 (3.21-3.84)** |

aHR, adjusted hazard ratio; CI, confidence interval.

Models were adjusted for disease history (ischemic heart disease, cerebrovascular disease, heart failure, chronic hepatitis B/C, liver cirrhosis, fatty liver, chronic obstructive pulmonary disease, pneumonia, tuberculosis, haematuria, and cystitis) and medication use (anti-diabetic drugs, aspirin, non-steroidal anti-inflammatory drugs, anti-coagulants, anti-platelets, statins, and anti-hypertensive drugs).

Table S3. Sensitivity analysis - Factors associated with site-specific cancer risk without excluding patients with a very short follow-up period.

ii) Diabetes

|  | **Diabetes** | | | | | | |
| --- | --- | --- | --- | --- | --- | --- | --- |
|  | **Colon & Rectum** | **Liver** | **Pancreas** | **Bladder** | **Kidney** | **Stomach** | **Lung** |
| **Factor** | **aHR (95%CI)** | **aHR (95%CI)** | **aHR (95%CI)** | **aHR (95%CI)** | **aHR (95%CI)** | **aHR (95%CI)** | **aHR (95%CI)** |
| Metabolic factors |  |  |  |  |  |  |  |
| Fasting glucose, every 1 mmol/L increase | 1.02 (1.00-1.04) | 1.02 (1.00-1.05) | **1.10 (1.07-1.13)** | 0.98 (0.93-1.03) | 0.96 (0.90-1.03) | 1.02 (0.98-1.06) | 0.99 (0.97-1.02) |
| Low-density lipoprotein cholesterol, every 1 mmol/L increase | 0.97 (0.92-1.03) | **0.82 (0.76-0.89)** | **0.81 (0.72-0.90)** | 1.00 (0.87-1.15) | 1.05 (0.89-1.24) | 1.00 (0.89-1.13) | 1.03 (0.96-1.09) |
| High-density lipoprotein cholesterol, every 1 mmol/L increase | 0.95 (0.81-1.11) | **1.31 (1.09-1.58)** | **0.67 (0.51-0.88)** | 1.09 (0.76-1.57) | 0.71 (0.44-1.15) | **0.43 (0.31-0.62)** | 0.87 (0.73-1.03) |
| Triglycerides, every 1 mmol/L increase | 0.99 (0.95-1.04) | **0.80 (0.73-0.87)** | **0.76 (0.68-0.85)** | 1.05 (0.98-1.12) | 0.91 (0.79-1.05) | 0.90 (0.81-1.00) | 0.96 (0.91-1.01) |
| Alanine transaminase, every 20 U/L increase | **0.93 (0.90-0.97)** | **1.02 (1.02-1.03)** | **1.02 (1.02-1.03)** | 1.01 (0.98-1.04) | 1.00 (0.97-1.04) | 1.01 (0.99-1.03) | 0.98 (0.95-1.01) |
| Common risk factors |  |  |  |  |  |  |  |
| Male | **1.64 (1.47-1.83)** | **2.14 (1.83-2.51)** | 1.08 (0.90-1.31) | **2.87 (2.11-3.91)** | **1.59 (1.15-2.19)** | **1.41 (1.13-1.76)** | **1.22 (1.07-1.39)** |
| Age, every 10-year increase | **1.65 (1.58-1.73)** | **1.50 (1.41-1.59)** | **1.65 (1.53-1.79)** | **1.90 (1.70-2.13)** | 1.05 (0.93-1.19) | **1.83 (1.67-2.00)** | **1.81 (1.72-1.91)** |
| Ever smoker | **1.42 (1.28-1.59)** | **1.62 (1.42-1.86)** | **1.35 (1.11-1.65)** | **1.98 (1.55-2.52)** | 1.36 (1.00-1.86) | **1.38 (1.11-1.71)** | **3.47 (3.09-3.90)** |

aHR, adjusted hazard ratio; CI, confidence interval.

Models were adjusted for disease history (ischemic heart disease, cerebrovascular disease, heart failure, chronic hepatitis B/C, liver cirrhosis, fatty liver, chronic obstructive pulmonary disease, pneumonia, tuberculosis, haematuria, and cystitis) and medication use (anti-diabetic drugs, aspirin, non-steroidal anti-inflammatory drugs, anti-coagulants, anti-platelets, statins, and anti-hypertensive drugs).

iii) No Diabetes

|  | **No Diabetes** | | | | | | |
| --- | --- | --- | --- | --- | --- | --- | --- |
|  | **Colon & Rectum** | **Liver** | **Pancreas** | **Bladder** | **Kidney** | **Stomach** | **Lung** |
| **Factor** | **aHR (95%CI)** | **aHR (95%CI)** | **aHR (95%CI)** | **aHR (95%CI)** | **aHR (95%CI)** | **aHR (95%CI)** | **aHR (95%CI)** |
| Metabolic factors |  |  |  |  |  |  |  |
| Fasting glucose, every 1 mmol/L increase | **1.14 (1.05-1.24)** | **1.20 (1.07-1.35)** | 1.13 (0.84-1.51) | 0.83 (0.64-1.06) | 0.87 (0.65-1.17) | **1.26 (1.11-1.44)** | **1.11 (1.02-1.21)** |
| Low-density lipoprotein cholesterol, every 1 mmol/L increase | **0.92 (0.85-0.98)** | **0.78 (0.68-0.88)** | 0.96 (0.76-1.21) | 1.06 (0.89-1.25) | 0.95 (0.77-1.17) | 0.89 (0.77-1.04) | 0.95 (0.89-1.03) |
| High-density lipoprotein cholesterol, every 1 mmol/L increase | 0.85 (0.70-1.04) | **0.66 (0.47-0.92)** | 1.14 (0.67-1.96) | 0.87 (0.54-1.39) | **0.47 (0.26-0.87)** | 0.83 (0.56-1.21) | 0.92 (0.76-1.11) |
| Triglycerides, every 1 mmol/L increase | 1.03 (0.96-1.11) | **0.66 (0.54-0.81)** | 0.85 (0.61-1.18) | 1.03 (0.84-1.25) | 0.89 (0.69-1.15) | 0.90 (0.74-1.09) | 1.00 (0.92-1.09) |
| Alanine transaminase, every 20 U/L increase | 0.99 (0.96-1.02) | **1.03 (1.02-1.04)** | 1.02 (0.98-1.07) | 1.00 (0.96-1.05) | 0.98 (0.89-1.08) | 0.98 (0.92-1.05) | 0.97 (0.94-1.01) |
| Common risk factors |  |  |  |  |  |  |  |
| Male | **1.48 (1.28-1.72)** | **2.49 (1.88-3.29)** | 1.41 (0.91-2.17) | **3.30 (2.12-5.14)** | **1.72 (1.10-2.69)** | **1.69 (1.25-2.29)** | 1.11 (0.95-1.31) |
| Age, every 10-year increase | **1.71 (1.62-1.81)** | **1.54 (1.41-1.69)** | **1.74 (1.45-2.08)** | **2.12 (1.83-2.45)** | **1.22 (1.05-1.42)** | **1.74 (1.56-1.95)** | **1.62 (1.53-1.72)** |
| Ever smoker | **1.31 (1.13-1.52)** | **1.68 (1.33-2.12)** | 1.24 (0.76-2.02) | **2.19 (1.59-3.02)** | 1.44 (0.96-2.15) | **1.38 (1.03-1.85)** | **3.63 (3.14-4.18)** |

aHR, adjusted hazard ratio; CI, confidence interval.

Models were adjusted for disease history (ischemic heart disease, cerebrovascular disease, heart failure, chronic hepatitis B/C, liver cirrhosis, fatty liver, chronic obstructive pulmonary disease, pneumonia, tuberculosis, haematuria, and cystitis) and medication use (aspirin, non-steroidal anti-inflammatory drugs, anti-coagulants, anti-platelets, statins, and anti-hypertensive drugs).

Table S4. Sensitivity analysis - Factors associated with site-specific cancer risk after excluding patients with a follow-up period of less than one year.

i) Overall

|  | **Overall** | | | | | | |
| --- | --- | --- | --- | --- | --- | --- | --- |
|  | **Colon & Rectum** | **Liver** | **Pancreas** | **Bladder** | **Kidney** | **Stomach** | **Lung** |
| **Factor** | **aHR (95%CI)** | **aHR (95%CI)** | **aHR (95%CI)** | **aHR (95%CI)** | **aHR (95%CI)** | **aHR (95%CI)** | **aHR (95%CI)** |
| Metabolic factors |  |  |  |  |  |  |  |
| Diabetes | 1.05 (0.88-1.25) | **1.49 (1.17-1.90)** | **1.82 (1.21-2.73)** | 1.52 (0.99-2.33) | 1.08 (0.64-1.81) | 1.00 (0.70-1.44) | 1.13 (0.93-1.37) |
| Fasting glucose, every 1 mmol/L increase | **1.03 (1.01-1.05)** | **1.04 (1.02-1.07)** | 1.04 (0.99-1.08) | 0.97 (0.92-1.03) | 0.97 (0.91-1.04) | **1.05 (1.00-1.09)** | 1.00 (0.98-1.02) |
| Low-density lipoprotein cholesterol, every 1 mmol/L increase | 1.01 (0.96-1.06) | **0.78 (0.72-0.84)** | **0.86 (0.77-0.97)** | 1.05 (0.93-1.18) | 1.01 (0.87-1.17) | 1.03 (0.93-1.14) | 1.00 (0.95-1.06) |
| High-density lipoprotein cholesterol, every 1 mmol/L increase | 0.98 (0.86-1.11) | 1.15 (0.96-1.38) | 0.83 (0.61-1.13) | 1.09 (0.80-1.48) | 0.66 (0.43-1.01) | **0.71 (0.53-0.93)** | 0.94 (0.81-1.08) |
| Triglycerides, every 1 mmol/L increase | **1.03 (1.01-1.06)** | **0.80 (0.73-0.87)** | **0.87 (0.78-0.98)** | 1.03 (0.95-1.12) | 0.91 (0.79-1.04) | 0.96 (0.88-1.06) | 0.99 (0.94-1.03) |
| Alanine transaminase, every 20 U/L increase | 0.99 (0.96-1.01) | **1.02 (1.02-1.03)** | 0.97 (0.92-1.03) | 1.01 (0.97-1.04) | 0.96 (0.88-1.04) | 1.01 (0.98-1.03) | 0.98 (0.96-1.01) |
| Common risk factors |  |  |  |  |  |  |  |
| Male | **1.61 (1.46-1.77)** | **2.14 (1.84-2.49)** | **1.29 (1.04-1.61)** | **3.26 (2.47-4.32)** | **1.62 (1.21-2.18)** | **1.51 (1.24-1.84)** | **1.17 (1.04-1.30)** |
| Age, every 10-year increase | **1.70 (1.63-1.76)** | **1.50 (1.42-1.59)** | **1.67 (1.53-1.82)** | **1.96 (1.78-2.16)** | 1.11 (0.99-1.24) | **1.78 (1.65-1.93)** | **1.77 (1.70-1.85)** |
| Ever smoker | **1.37 (1.24-1.50)** | **1.69 (1.48-1.92)** | **1.45 (1.16-1.80)** | **1.89 (1.53-2.33)** | 1.27 (0.96-1.68) | **1.36 (1.12-1.66)** | **3.68 (3.33-4.07)** |

aHR, adjusted hazard ratio; CI, confidence interval.

Models were adjusted for disease history (ischemic heart disease, cerebrovascular disease, heart failure, chronic hepatitis B/C, liver cirrhosis, fatty liver, chronic obstructive pulmonary disease, pneumonia, tuberculosis, haematuria, and cystitis) and medication use (anti-diabetic drugs, aspirin, non-steroidal anti-inflammatory drugs, anti-coagulants, anti-platelets, statins, and anti-hypertensive drugs).

Table S4. Sensitivity analysis - Factors associated with site-specific cancer risk after excluding patients with a follow-up period of less than one year.

ii) Diabetes

|  | **Diabetes** | | | | | | |
| --- | --- | --- | --- | --- | --- | --- | --- |
|  | **Colon & Rectum** | **Liver** | **Pancreas** | **Bladder** | **Kidney** | **Stomach** | **Lung** |
| **Factor** | **aHR (95%CI)** | **aHR (95%CI)** | **aHR (95%CI)** | **aHR (95%CI)** | **aHR (95%CI)** | **aHR (95%CI)** | **aHR (95%CI)** |
| Metabolic factors |  |  |  |  |  |  |  |
| Fasting glucose, every 1 mmol/L increase | 1.02 (1.00-1.04) | **1.03 (1.00-1.06)** | 1.04 (1.00-1.09) | 0.98 (0.92-1.04) | 0.97 (0.91-1.05) | 1.04 (0.99-1.08) | 1.00 (0.97-1.02) |
| Low-density lipoprotein cholesterol, every 1 mmol/L increase | 1.02 (0.96-1.09) | **0.79 (0.72-0.86)** | **0.84 (0.73-0.95)** | 1.00 (0.86-1.17) | 1.07 (0.89-1.28) | 1.06 (0.93-1.20) | 1.02 (0.95-1.09) |
| High-density lipoprotein cholesterol, every 1 mmol/L increase | 1.04 (0.88-1.23) | **1.35 (1.10-1.66)** | 0.75 (0.53-1.07) | 1.22 (0.81-1.84) | 0.72 (0.41-1.25) | **0.52 (0.35-0.76)** | 0.92 (0.76-1.12) |
| Triglycerides, every 1 mmol/L increase | 1.02 (0.99-1.06) | **0.82 (0.75-0.90)** | **0.87 (0.77-0.98)** | 1.04 (0.96-1.12) | 0.92 (0.79-1.08) | 0.94 (0.84-1.05) | 0.98 (0.92-1.03) |
| Alanine transaminase, every 20 U/L increase | **0.96 (0.93-1.00)** | **1.02 (1.02-1.03)** | 0.96 (0.90-1.04) | 1.01 (0.97-1.05) | 0.92 (0.81-1.05) | 1.01 (0.99-1.04) | 0.98 (0.95-1.01) |
| Common risk factors |  |  |  |  |  |  |  |
| Male | **1.65 (1.46-1.87)** | **2.05 (1.72-2.44)** | 1.27 (0.99-1.61) | **3.17 (2.25-4.46)** | **1.53 (1.06-2.21)** | **1.45 (1.13-1.85)** | **1.22 (1.05-1.41)** |
| Age, every 10-year increase | **1.66 (1.58-1.74)** | **1.50 (1.40-1.60)** | **1.66 (1.50-1.83)** | **1.92 (1.70-2.17)** | 1.05 (0.90-1.21) | **1.86 (1.68-2.06)** | **1.83 (1.73-1.94)** |
| Ever smoker | **1.45 (1.29-1.64)** | **1.64 (1.41-1.92)** | **1.48 (1.17-1.89)** | **1.85 (1.42-2.41)** | 1.41 (0.99-2.00) | **1.31 (1.02-1.67)** | **3.64 (3.20-4.14)** |

aHR, adjusted hazard ratio; CI, confidence interval.

Models were adjusted for disease history (ischemic heart disease, cerebrovascular disease, heart failure, chronic hepatitis B/C, liver cirrhosis, fatty liver, chronic obstructive pulmonary disease, pneumonia, tuberculosis, haematuria, and cystitis) and medication use (anti-diabetic drugs, aspirin, non-steroidal anti-inflammatory drugs, anti-coagulants, anti-platelets, statins, and anti-hypertensive drugs).

iii) No Diabetes

|  | **No Diabetes** | | | | | | |
| --- | --- | --- | --- | --- | --- | --- | --- |
|  | **Colon & Rectum** | **Liver** | **Pancreas** | **Bladder** | **Kidney** | **Stomach** | **Lung** |
| **Factor** | **aHR (95%CI)** | **aHR (95%CI)** | **aHR (95%CI)** | **aHR (95%CI)** | **aHR (95%CI)** | **aHR (95%CI)** | **aHR (95%CI)** |
| Metabolic factors |  |  |  |  |  |  |  |
| Fasting glucose, every 1 mmol/L increase | **1.11 (1.02-1.22)** | **1.25 (1.11-1.41)** | 0.87 (0.60-1.27) | 0.78 (0.58-1.03) | 0.83 (0.59-1.17) | **1.24 (1.06-1.45)** | 1.07 (0.97-1.18) |
| Low-density lipoprotein cholesterol, every 1 mmol/L increase | 0.99 (0.91-1.07) | **0.76 (0.66-0.88)** | 1.00 (0.78-1.27) | 1.12 (0.93-1.34) | 0.92 (0.72-1.17) | 1.00 (0.85-1.18) | 0.99 (0.91-1.07) |
| High-density lipoprotein cholesterol, every 1 mmol/L increase | 0.93 (0.76-1.14) | 0.82 (0.57-1.18) | 1.14 (0.65-2.01) | 0.87 (0.52-1.46) | 0.58 (0.30-1.13) | 1.07 (0.71-1.61) | 0.96 (0.78-1.19) |
| Triglycerides, every 1 mmol/L increase | **1.08 (1.02-1.15)** | **0.70 (0.57-0.87)** | 0.87 (0.62-1.22) | 0.97 (0.76-1.24) | 0.85 (0.63-1.17) | 1.02 (0.85-1.21) | 1.02 (0.93-1.11) |
| Alanine transaminase, every 20 U/L increase | 1.00 (0.98-1.03) | **1.03 (1.02-1.04)** | 1.00 (0.92-1.09) | 1.01 (0.95-1.06) | 0.99 (0.90-1.09) | 0.98 (0.90-1.06) | 0.99 (0.95-1.03) |
| Common risk factors |  |  |  |  |  |  |  |
| Male | **1.51 (1.29-1.78)** | **2.31 (1.69-3.16)** | 1.47 (0.94-2.32) | **3.59 (2.19-5.87)** | **1.79 (1.09-2.94)** | **1.63 (1.17-2.28)** | 1.08 (0.91-1.30) |
| Age, every 10-year increase | **1.74 (1.63-1.85)** | **1.52 (1.37-1.69)** | **1.75 (1.45-2.11)** | **2.02 (1.73-2.37)** | **1.22 (1.02-1.45)** | **1.65 (1.46-1.87)** | **1.69 (1.59-1.80)** |
| Ever smoker | **1.30 (1.10-1.53)** | **1.89 (1.45-2.45)** | 1.22 (0.73-2.03) | **1.96 (1.38-2.78)** | 1.04 (0.65-1.68) | **1.48 (1.07-2.05)** | **3.80 (3.24-4.45)** |

aHR, adjusted hazard ratio; CI, confidence interval.

Models were adjusted for disease history (ischemic heart disease, cerebrovascular disease, heart failure, chronic hepatitis B/C, liver cirrhosis, fatty liver, chronic obstructive pulmonary disease, pneumonia, tuberculosis, haematuria, and cystitis) and medication use (aspirin, non-steroidal anti-inflammatory drugs, anti-coagulants, anti-platelets, statins, and anti-hypertensive drugs).

Table S5. Sensitivity analysis - Association between diabetes and site-specific cancer risk after excluding a diagnosis of diabetes within the first year of the prescription records.

|  | **Diabetes vs No Diabetes** |
| --- | --- |
| **Cancer site** | **aHR (95% CI)** |
| Colon & Rectum | 1.12 (0.96-1.31) |
| Liver | **1.47 (1.19-1.83)** |
| Pancreas | **2.47 (1.77-3.44)** |
| Bladder | 1.46 (0.99-2.17) |
| Kidney | 1.07 (0.68-1.68) |
| Stomach | 1.14 (0.83-1.59) |
| Lung | 1.00 (0.84-1.19) |

aHR, adjusted hazard ratio; CI, confidence interval.

Models were adjusted for age, sex, smoking, laboratory measurements (fasting glucose, low-density lipoprotein cholesterol, high-density lipoprotein cholesterol, triglycerides, and alanine transaminase), disease history (ischemic heart disease, cerebrovascular disease, heart failure, chronic hepatitis B/C, liver cirrhosis, fatty liver, chronic obstructive pulmonary disease, pneumonia, tuberculosis, haematuria, and cystitis) and medication use (anti-diabetic drugs, aspirin, non-steroidal anti-inflammatory drugs, anti-coagulants, anti-platelets, statins, and anti-hypertensive drugs).
